# Transport IVF-ICSI: Results of a 25-year experience

**DOI:** 10.5935/1518-0557.20180026

**Published:** 2018

**Authors:** Fernanda G.E. Raffo, Jorge Blaquier

**Affiliations:** 1 FERTILAB Centro Médico. Buenos Aires, Argentina

**Keywords:** Transport Assisted Reproduction, IVF, ICSI

## Abstract

**Objective:**

This report presents a summary of the outcomes achieved at ART center FERTILAB in Buenos Aires, Argentina, with transport IVF-ICSI for 25 years (1990-2014).

**Methods:**

This report included all patients submitted to oocyte retrieval for IVF-ICSI whose oocytes were transported from sites of aspiration located 0.5-58 kilometers away from the central laboratory. The numeric data herein reported were taken from annual reports submitted by our institution (Fertilab) and, for purposes of comparison, by all Argentinian centers to the Latin American Registry of Assisted Reproduction (RLA) within the same time period.

**Results:**

From 1990 to 2014, 5091 aspirations followed by oocyte transport were performed in our center, resulting in 1258 pregnancies after fresh embryo transfers. The mean pregnancy/aspiration rate for the 25-year period was 24.71%. To validate the efficacy of our transport system, our results were compared to the outcomes of Argentinian centers reporting to RLA Argentina in the period ranging from 1990 to 2014. A total of 79,062 aspirations were performed, yielding 20,047 pregnancies and a pregnancy/aspiration rate of 25.36%. Delivery/aspirations rates were 15.34% for Fertilab patients and 14.79% for RLA Argentina centers.

**Conclusion:**

The results showed that the differences in clinical outcomes between our center and the bulk of Argentinian centers were not statistically significant, indicating that oocyte transport does not decrease the effectiveness of IVF-ICSI and might be advantageous under certain circumstances.

## INTRODUCTION

This report presents a summary of the outcomes achieved at ART center FERTILAB in Buenos Aires, Argentina, with transport IVF-ICSI for 25 years (1990-2014). When we started our activities in 1984, oocyte retrieval was performed laparoscopically, requiring a fully equipped operating room and support services not available at our center. To solve this problem, we followed the lead set by Plachot *et al*. (1984) and Zeilmaker *et al.* (1987) regarding the transportation of oocytes from satellite clinics under controlled conditions. This approach proved successful in our clinic, and despite the simplification introduced by ultrasound guided transvaginal oocyte aspiration, we kept using transport IVF-ICSI for our own cases and patients seen in other clinics.

The reasons to adopt this policy were:

- It allowed independent physicians and doctors working at fertility clinics not equipped with IVF laboratories to offer high quality reproductive care to their patients. Ovarian stimulation monitoring and oocyte harvesting *in situ*, associated with transport IVF/ICSI, alleviated some of the psychological, physical, and financial burdens of patients seen in remote institution, including travel and accommodation expenses, and loss of work.
- In addition to enabling a friendlier patient environment, this approach reduced the costs of setting up an IVF laboratory for smaller groups, lowering prices and making ART available to a larger portion of the population.
- In clinics with limited surgical resources such as ours, having oocyte retrievals performed at fully equipped medical institutions enabled us to effectively cope with emergencies, fortunately a very rare event, and offer peace of mind to patients and physicians alike.
- The large number of procedures performed in our institution improved the consistency of laboratory methodology and enhanced the cost-effectiveness of the laboratory.

In 1998, our group published a report (Alfonsín *et al*., 1998) comparing the results of 575 cases of transport IVF-ICSI with oocytes aspirated at four different large hospitals in the city of Buenos Aires to the outcomes of 60 IVF-ICSI cases performed *in situ* at one of these hospitals. The results demonstrated that, in our hands, oocyte transportation did not lead to inferior ART treatment outcomes, with both groups yielding similar results. In cases of necessity, embryos have also been transported back to the facilities where they were originally retrieved without detriment to their capacity of originating pregnancies, as shown in this report. By force of Law, the entire Argentinian population has had access to ART free of charge since 2014. For unexplained reasons, however, a regulation issued by the Ministry of Health banned the transportation of fresh oocytes, bringing our experience to an abrupt end in late 2015.

This report aimed to share the outcomes of our long experience with transport IVF-ICSI since it might prove useful to centers located in other countries and subject to different circumstances, and as it makes ART effective, safe and less costly.

## MATERIALS AND METHODS

In transport IVF-ICSI, oocyte retrieval is performed in a remote facility and oocytes are transported to an IVF clinic immediately after retrieval.

This report included only patients offered oocyte retrieval and transport IVF-ICSI from 1990 to late 2014 (5091 from our center). This time period was chosen because since 1990 the annual reports issued to the Latin American Registry of Assisted Reproduction (RLA) have contained more reliable and detailed data from all participating centers. Detailed data from our institution (Fertilab) were compared to the data reported by all Argentinian centers to the RLA (RLA Argentina).

Controlled ovarian hyperstimulation procedures have varied along time. Initially, urinary gonadotropins and clomiphene citrate were used (Hillier *et al*., 1985; Messinis *et al*., 1986); then in 1992, the agonist long protocol combined with purified FSH and HMG took over (Porter *et al*., 1984; Frydman *et al*., 1988); in 2007, most cycles were done with antagonist and recombinant FSH and urinary HMG (Olivennes *et al*., 2002). These drugs were administered following well-established protocols adapted to the needs of each patient. Oocyte retrieval was performed by ultrasound guided vaginal aspiration under mild sedation. Cumulus-oocyte complexes were isolated *in situ* using a portable modified neonatal incubator, (Alfonsín *et al.*, 1998), washed in transport media (modified HTF from Irvine Scientific, Santa Ana, CA), supplemented with 10% Human Serum Albumin (Origio, Malv, Denmark), placed in a tightly capped tube (Falcon 2003 BD Biosciences, Two Oak Park Bedford, MA 01730 USA), and stored in a portable incubator (Portable Incubator G95 K-SYSTEMS: Kivex Biotec A/S Klintehøj Vænge 3-5, DK- 3460 Birkerød) at 37°C for transport. In every instance the time elapsed between retrieval and insemination or injection was recorded. The distance of transport ranged from 0.5 to 58 kilometers.

In all other procedures, oocyte processing, insemination or injection for ICSI, embryo culture, and embryo transfer were performed at the central laboratory following published procedures (Alfonsín *et al.*, 1998). In a few cases (singled out in the Results section), the embryos were transported back to the site of retrieval for transfer (two-way transport). The same transport medium and portable incubator were used in these cases.

Until 2006, embryo cryopreservation was performed using the slow freezing technique described by Mandelbaum *et al*. (1987) and Gardner *et al*. (2003), using a CL-8800 temperature controller, cryochamber and cryobath (CryoLogic Pty. 1/2-6 Apollo Ct, Blackburn VIC 3130, Australia). Since then vitrification was adopted following the procedure described by Kuwayama *et al*. (2005). The RLA started capturing data for frozen embryo transfers in 1995.

Statistical analysis was performed on software package GraphPad INSTAT version 4. The chi-square test was used to compare groups for a 95% confidence interval. Statistical significance was assigned to differences with *p*<0.05

### Quality control of the transport procedure

**Figure 1:**
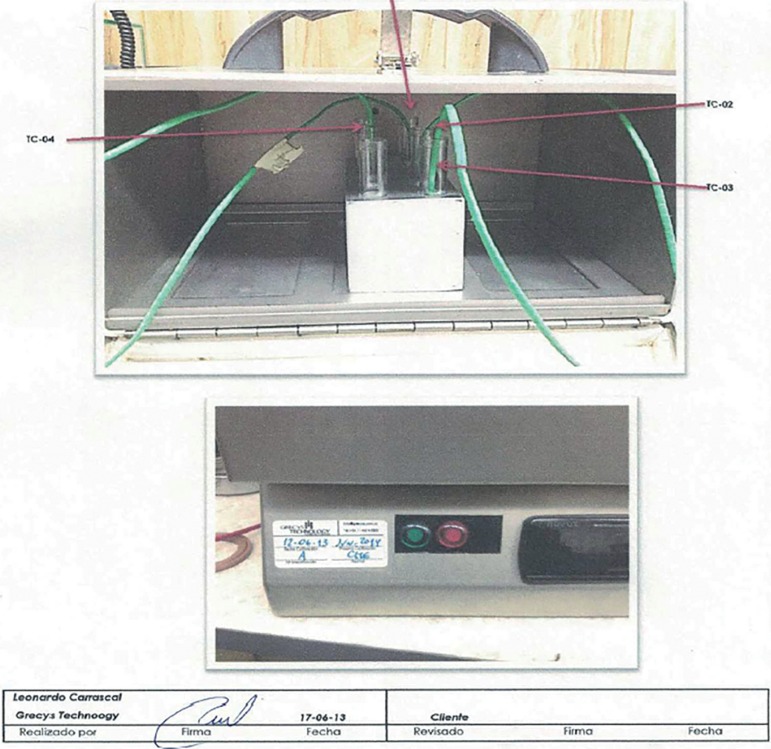
Temperature control in portable incubator

**Figure 2:**
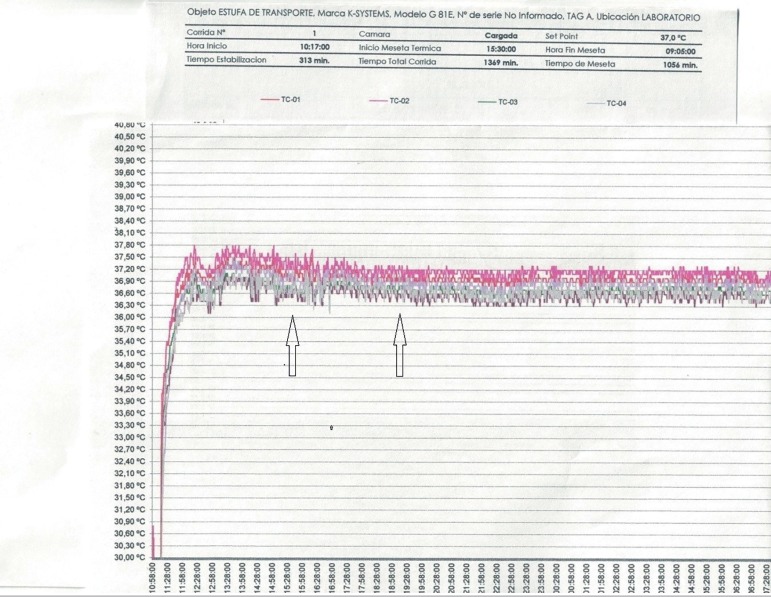
24-hour temperature control test of portable incubator. The incubator was connected to a 220-volt power outlet except for a period of four hours (area between arrows), during which the incubator was running on batteries

The adoption of transport IVF-ICSI in our clinic made it clear from the outset that high levels of quality control and quality assurance were needed for the continuing success of this approach. With pH controlled by HEPES buffered medium, our main concern shifted to temperature stability for prolonged periods of time. Our transport incubators were each fitted with 315C PTC model certified contact thermometers (PTC Instruments, Los Angeles, CA, USA), and temperatures at the time of departure and arrival were recorded. Once a year each transport incubator was tested for 24 hours for temperature control, first connected to a power outlet (220 V) for 20 hours and then running on batteries for four hours. One of these tests, showing how temperatures are measured and test results, is illustrated in Figures 1 and 2. A report published by our group (Alfonsín *et al*., 1998) found that the mean time elapsed between oocyte aspiration and culture was 110 minutes, and further described that only seven of 5300 (0.13%) cumulus-oocyte complexes were lost during transport. Delays were recorded in a few cases (two to four hours of transport time), either due to vehicle breakdown or roadblocks, which apparently did not cause deleterious effects on the oocytes.

## RESULTS

From 1990 to 2014, 5091 aspirations followed by oocyte transport were performed in our center, resulting in 1258 pregnancies after fresh embryo transfers. The mean pregnancy/aspiration rate for the 25-year period was 24.71%, with rates ranging from 19.6% to 43.88%.

To validate the efficacy of our transport system, our results were compared to the outcomes of Argentinian centers reporting to RLA Argentina in the period ranging from 1990 to 2014 (Table 1). The results revealed that the differences in clinical outcomes between our center and the bulk of Argentinian centers were not statistically significant.

**Table 1:** Oocyte retrievals, pregnancies, and deliveries at Fertilab and all Argentinian centers reporting to the RLA (RLA Argentina) after fresh embryo transfers, 1990-2014.

Clinical outcome	Fertilab	RLA Argentina	*p*
Oocyte retrievals	5091	79062	
Clinical pregnancies	1258	20047	
Pregnancy/Retrieval rate %	24.71	25.36	0.31
Deliveries	781	11693	
Delivery/Retrieval rate %	15.34	14.79	0.24

The results were segregated by patient age (<35; 35-39; and >39 years of age). Table 2 shows the results and comparisons with RLA Argentina. Performance improved in the 25 years examined in this report as experience grew. The pregnancy/aspiration rate was 17.67% from 1990 to 1995; 25.25% from 1996 to 2000; 27.04% from 2001 to 2005; and 29.21% from 2006 to 2010. These results compared favorably to the outcomes observed in RLA Argentina, although they were not significantly different (17.61%; 24.66%; 24.24%; and 29.2%, respectively). Similarly, the number of fresh embryos transferred decreased as pregnancy rates increased. The mean number of embryos transferred at Fertilab was 3.63 in 1995-99; 2.61 in 2000-2004; 1.98 in 2005-2009; and 2.12 in 2010-2013. The corresponding numbers for RLA Argentina were 3.26; 2.72; 2.34; and 2.07. The implantation rate in 1995-2010 was 13.24% at Fertilab and 14.28% at RLA Argentina.

**Table 2:** Pregnancy rates at Fertilab and all Argentinian centers reporting to the RLA (RLA Argentina) segregated by patient age, 1995-2010.

Center and age group	Transfers	Pregnancies	Pregnancies %
**FERTILAB**			
<35 years 35-39 years >39 years	1019 873 472	389 269 85	38.17 30.81 18.01
**RLA Argentina**			
<35 years 35-39 years >39 years	21495 18895 8739	7293 5287 1353	33.93 27.98 15.48

Table 3 shows the results of frozen embryo transfers (FET) in 1995-2014 and the comparison against RLA Argentina. Again, the analysis of results showed that the differences between the two groups were not statistically significant.

**Table 3:** Comparison of results of frozen embryo transfers between Fertilab and all Argentinian centers reporting to the RLA (RLA Argentina), 1995-2014.

Clinical outcome	Fertilab	RLA Argentina	*p*
Embryo transfers	1690	16897	
Clinical pregnancies	432	4294	
Pregnancy/transfer rate %	25.56	25.41	0.91
Deliveries	312	3209	
Deliveries/transfer rate %	18.46	18.99	0.61
Multiple pregnancies	50	577	
Multiple pregnancy rate %	16.03	17.98	0.85

Table 4 shows the cumulative pregnancy and delivery rates per patient after the transfer of fresh and frozen embryos, yielded from a single retrieval procedure between 1995 and 2014. Our results were not statistically different from the outcomes at RLA Argentina.

**Table 4:** Cumulative (fresh + frozen embryos) pregnancies and deliveries. Period 1995-2014.

Clinical Outcome	Fertilab	RLA Argentina	*p*
Oocyte Retrievals	5058	83257	
Clinical pregnancies	1573	24681	
Pregnancy/Retrieval rate %	31.10	29.64	0.87
Deliveries	856	15199	
Delivery/Retrieval rate %	16.92	18.26	1

In a few instances two-way transport was performed for a clinic located 58 kilometers from our central laboratory. After pickup, the oocytes were transported to the central laboratory and at the time of transfer the embryos were returned to the site of retrieval for the transfer procedure. The results were as follows: 43 retrievals yielded a mean of 10 oocytes per retrieval, 7.27 of which were mature; ICSI yielded 252 embryos and a fertilization rate of 80.5%. Upon embryo transfer, 18 pregnancies were initiated resulting in a pregnancy per retrieval rate of 44%. In this series, no oocyte or embryo was lost during transport. We had trouble recording deliveries in this group, since a substantial number of patients came from remote parts of the country and were lost to follow-up; hence, the data on deliveries was not given.

Our clinic started an oocyte donation program in 2010. Donor oocytes were transported from the site of aspiration to the central laboratory. In 2015, a total of 467 recipients had been prepared and 435 had received a mean of 1.96 embryo. Two hundred and fourteen clinical pregnancies were initiated (49.2% pregnancies per transfer) with 130 deliveries (29.89% delivery/transfer rate), 18.98% of which were multiple births. The corresponding numbers for RLA Argentina were 6253 transfers, 2877 pregnancies (46.01% pregnancies/transfer), and 2025 deliveries (32.38% deliveries/transfer), 24.15% of which were multiple births.

## DISCUSSION

This report presents a summary of the outcomes achieved at our center with transport IVF-ICSI for 25 years. Many were the merits of transport IVF-ICSI in our center: it broadened the base of physicians using our services; it allowed patients to undergo treatment at the institution of their choice; and it decreased costs, as many clinics were able to share one central laboratory. This report resorted to data collected by the Latin American Registry of Assisted Reproduction, an institution collecting data from individual centers in many Latin American countries since 1990. Detailed data from our center and from all Argentinian centers reporting to the RLA were used in this report for purposes of comparison. Using these data instead of our own records provided homogeneity to the sample and validity to the comparison against other regional centers.

The main concern when we first started with transport IVF-ICSI was that transportation might impair the success rate of ART procedures. An earlier publication (Alfonsín *et al*., 1998) enrolling a smaller number of cases compared the outcomes of procedures performed *in situ* versus procedures performed with transported oocytes and found no significant differences in the number of pregnancies attained.

The present results confirmed, in a larger scale, that oocyte transport did not decrease the effectiveness of ART procedures after either fresh or frozen embryo transfers. Two-way transportation, with embryos being returned to the site of oocyte aspiration, also yielded excellent results. Our experience is coincident with that reported mainly in the Netherlands (Jansen *et al*., 1986; Roest *et al*., 1995), a country in which this practice is current and extensive. For example, a 2004 national prospective study of pregnancy chances after IVF-ICSI in the Netherlands (Lintsen *et al*., 2007) included 13 conventional IVF centers and 23 transport IVF clinics.

Transport IVF is used in other countries in Europe (Kingsland *et al*., 1992; De Sutter *et al*., 1996; Qureshi *et al*., 1997), Canada (Buckett *et al*., 1999) and Japan (Takanashi *et al*., 2004). Fresh embryo transport is also available in the USA (Langley *et al*., 2001), Ukraine (Levron *et al.*, 2014), and Japan (Takanashi *et al.,* 2005), with satisfying results.

In summary, transport of oocytes is a successful approach to ART in several countries.
